# Effects of Melanocortin 1 Receptor Agonists in Experimental Nephropathies

**DOI:** 10.1371/journal.pone.0087816

**Published:** 2014-01-30

**Authors:** Annika Lindskog Jonsson, Anna Granqvist, Johannes Elvin, Martin E. Johansson, Börje Haraldsson, Jenny Nyström

**Affiliations:** 1 Department of Molecular and Clinical Medicine – Nephrology, Institute of Medicine, The Sahlgrenska Academy, University of Gothenburg, Gothenburg, Sweden; 2 Center for Molecular Pathology, Department of Laboratory Medicine, Lund University, SUS Malmö, Sweden; 3 Department of Physiology, Institute of Neuroscience and Physiology, The Sahlgrenska Academy, University of Gothenburg, Gothenburg, Sweden; Fondazione IRCCS Ospedale Maggiore Policlinico & Fondazione D’Amico per la Ricerca sulle Malattie Renali, Italy

## Abstract

Nephrotic syndrome, characterized by massive proteinuria, is caused by a large group of diseases including membranous nephropathy (MN) and focal segmental glomerulosclerosis (FSGS). Although the underlying mechanisms are beginning to unravel, therapy is unspecific and far from efficient. It has been suggested that adrenocorticotropic hormone (ACTH) has beneficial effects in patients with MN and possibly in other nephrotic diseases. We have previously reported that ACTH may act directly on podocytes through the melanocortin 1 receptor (MC1R). In the present study, we evaluate the effect of highly specific MC1R agonists in two different nephrotic disease models. Experimental MN: Passive Heymann nephritis (PHN) was induced in rats that were treated for four weeks with MS05, a selective MC1R agonist, or saline. The degree of albuminuria was significantly reduced over time and the effect was sustained one week after treatment withdrawal (p<0.05). Experimental FSGS: Based on a dose-response study, two doses of adriamycin were used for induction of nephropathy in Balb/c mice. Mice were treated with either a synthetic MC1R agonist (BMS-470539), with α-melanocyte stimulating hormone (α-MSH) or with saline. There was no beneficial effect of treatment. In summary, MC1R agonists reduce albuminuria and improve morphology in experimentally induced MN whereas they have no effect in experimental FSGS. The results illustrate the differences in these podocytopathies in terms of signaling mechanisms underlying proteinuria, and progression of disease.

## Introduction

The clinical entity nephrotic syndrome is caused by different morphological diseases such as membranous nephropathy (MN), minimal change disease (MCD), membranoproliferative glomerulonephritis (MPGN), and focal segmental glomerulosclerosis (FSGS). Recent studies have partially revealed the molecular mechanisms underlying these diseases. Thus, antibodies against phospholipase A2 seem to be involved in the pathogenesis of MN [1] as those to megalin are in passive Heymann nephritis in rats [2]. For patients with primary FSGS, soluble urokinase receptor (suPAR) has been reported to be elevated [3]. However, many questions remain [4]–[7], and treatment options are still far from specific or effective [8].

It was previously reported that treatment with adrenocorticotropic hormone (ACTH) had beneficial effects in patients with a wide range of nephrotic diseases [9], [10]. The effect was most prominent in patients with MN, but the treatment was also effective in some cases of MCD, diabetic nephropathy, MPGN and FSGS. The positive effects of ACTH on MN have been confirmed in a randomized controlled trial [11], albeit in low number of patients. In a recent study, we put forward a possible molecular mechanism behind the beneficial effects of the treatment [12]. Firstly, one of the ACTH receptors, melanocortin 1 receptor (MC1R), was expressed in the kidney, and more specifically in the podocytes. Secondly, MC1R agonists ameliorated the nephrotic disease in rats with passive Heymann nephritis (PHN), an experimental model of MN. Thus, ACTH and agonists of MC1R decreased proteinuria, reduced oxidative stress, and improved podocyte morphology.

Podocytes play a crucial role in the normal glomerulus and in the development of many glomerular diseases [13]. Although the onset of disease may vary, the resulting podocyte injury with foot process effacement and proteinuria is common in most proteinuric disorders such as human MN and FSGS. PHN in rats and adriamycin-induced nephropathy in mice are well-characterized models resembling human MN and FSGS respectively. PHN is characterized by immune deposits formed *in situ* in the glomerulus, triggered by antigen expression on the podocyte surface [14], mainly megalin [2]. Proteinuria reaches a peak level 14 days after disease induction and although immune deposit formation eventually ceases, proteinuria persists lifelong [14]. The adriamycin model is characterized by glomerulosclerosis, tubulo-interstitial inflammation and fibrosis [15], [16]. Podocytes [17]–[19], as well as endothelial cells and their surface layer [20], are damaged, leading to proteinuria.

In this study, we used different MC1R agonists to treat two experimental nephrotic diseases, MN and FSGS. The agonists used were: MS05, a selective peptide agonist for MC1R [21]; BMS-470539, a synthetic highly selective MC1R agonist [22]–[24]; and α-melanocyte stimulating hormone (α-MSH), an agonist for all melanocortin receptors 1–5 except type 2, which is selective only for ACTH.

The aim of this study was to evaluate the effects of MC1R agonists in two experimental nephrotic models, representing two different morphological diseases. Our hypothesis was that MC1R agonists would have similar beneficial effects in both MN and FSGS, and that they would ameliorate proteinuria.

## Materials and Methods

### Experimental Protocol

#### Animals

All animals had free access to standard food and water, and were housed in a room with a 12-hour dark-light cycle. Anesthesia was induced and maintained by inhalation of isoflurane (2–3% v/v, Schering-Plough, Stockholm, Sweden) mixed with air (∼1 L/min) in an isoflurane vaporizer (Ohmeda Isotec 5, Simtec engineering, Askim, Sweden). Temgesic® (0.1 ml/100 g body weight, Schering-Plough, Stockholm, Sweden) was given as a post-operative pain reliever in all small operative procedures. Gothenburg Ethical Board for Animal Experiments approved the study.

### Experimental MN – Passive Heymann Nephritis

The experiments were performed on male Sprague Dawley rats (Charles River, Germany) of initial body weight of 140–165 g. To induce passive Heymann nephritis (PHN), Anti-Fx1A IgG antibody, 30 mg/mL (Probetex Inc., San Antonio, TX), was slowly injected into the tail vein, 1.5 ml at day 0 and 0.5 ml at day 7. Instead of the PHN-inducing antibody, controls received sterile saline. At day 14–15, treatment with MS05 (custom-made peptide from Agrisera, Vännäs, Sweden), 100 µg/day was started, either via osmotic pump (Alzet® Osmotic Pumps, Cupertino, CA) placed subcutaneously in the neck or via subcutaneous injections. The osmotic pump was replaced after two weeks. Total treatment time was four weeks. After two additional weeks, the rats were sacrificed and the kidneys were harvested for further analyses. Weight was followed twice a week. Spot urine samples were collected twice a week and the analyses were based on 1–2 weekly observations per animal: controls, n = 5 (7–10 observations/week); untreated PHN, n = 14 (19–27 observations/week); MS05, n = 17 (19–33 observations/week).

### Experimental FSGS – Adriamycin-induced Nephrotic Syndrome

All experiments were performed on male BALB/c mice (Charles River, Germany) with an initial body weight of 22–26 g. To establish a stable nephropathy, a dose-response study using 5, 7, 8, 9 and 10 mg/kg (n = 5–19) was performed in order to find the optimal doses of adriamycin. Treatment, either BMS-470539 (30 µmol/kg, n = 18–19; synthetized by Enamine, Ukraine), or α-MSH (0.020 µmol/kg, n = 9; Sigma, St Louis, MO), diluted in DMSO or 1∶1 in water and PEG400 (Sigma, St Louis, MO), was started one day before adriamycin was given, either via osmotic pump (Alzet® Osmotic Pumps, Cupertino, CA) placed subcutaneously in the neck or via daily subcutaneous injections. Control mice, and mice given adriamycin with no subsequent treatment, received vehicle only.

At day 0, FSGS was induced by a single tail vein injection of 8 or 10 mg/kg adriamycin (doxorubicin hydrochloride, SigmaAldrich, St Louis, MO). An equal volume of saline was given to controls. To prevent weight loss due to low appetite, mice were given one intraperitoneal injection of a 2 mL glucose-electrolyte solution (16.7 g/L glucose in hypotonic 75 mM NaCl solution) on day 1 to 11. Body weight was recorded and spot urine samples collected daily. At day 7 to 12, the mice were sacrificed and the kidneys were harvested for further analyses.

### Urine Analyses

For rats, albumin was analyzed using the Rat Albumin Elisa Quantitation Kit (Bethyl Laboratories Inc., Montgomery, TX) and corrected for the urine creatinine concentration measured with the Jaffé reaction using a creatinine standard solution (Sigma, St Louis, MO). For mice, albumin was analyzed using the Albuwell M ELISA Kit and all urine samples were corrected for the urine creatinine concentration measured with the Creatinine Companion Kit (both from Exocell Inc., Philadelphia, PA). Optical density was measured on a Spectra max plus reader (Molecular Devices, Sunnyvale, CA).

### Blood Urea Nitrogen

Blood was taken from the mice through the caval vein at day 10 (8 mg/kg adriamycin) or day 8 (10 mg/kg adriamycin), centrifuged and plasma was immediately frozen and stored at −20°C. Blood urea nitrogen (BUN) was measured with QuantiChrom™ Urea Assay Kit (BioAssay Systems, Hayward, CA) according to manufacturer’s instructions.

### Real-time PCR

RNA was prepared from sieved isolated glomeruli using the Qiagen Mini Kit with DNAse digestion (Qiagen Nordic, Solna, Sweden). RNA quality was confirmed using the Standard Sensitivity Kit on Experion™ (Bio-Rad, Hercules, CA). RNA was quantified using a NanoDrop spectrophotometer (NanoDrop Technologies Inc., Wilmington, DE). Reverse transcription of RNA was performed using the High Capacity RNA-to-cDNA kit at a total volume of 20 µL (Applied Biosystems, Foster City, CA). 50 ng of sample cDNA was used to quantify the mRNA level of each target gene by real-time PCR on the ABI Prism 7900 Sequence Detection system (*Taq*Man, Applied Biosystems (ABI), Foster City, CA), as previously described [25]. The following primers and probes, all verified by ABI, were used to detect mRNA of the following genes: GAPDH: Mm99999915_g1 (mouse), Rn01775763_g1 (rat), Hs99999905_m1 (human); MC1R: Mm00434851_s1 (mouse), custom made GenBankID AB306978.1 (rat), Hs00267167_s1 (human). All samples were run in duplicates. RNA samples without performing reverse transcription were used as negative controls. The comparative ΔΔC_T_ method of relative quantification was used to calculate the differences in gene expression between the groups. GAPDH was used as endogenous control.

### Electron Microscopy

Immediately after blood sampling and sacrifice, the kidneys were collected for morphological analysis. The renal artery and vein were clamped and the kidney was fixed by subcapsular injection of Karnovsky’s fixative (2% paraformaldehyd and 2.5% glutaraldehyd in 0.05 M Na-cacodylate buffer, pH 7.2). The kidneys were cut into mm-slices and processed by standard procedure as previously described [26]. Slides were taken with a Leo 912AB Omega electron microscope (Leo Electron Microscopy Ltd., Cambridge, England) and examined in a blinded fashion by a pathologist as previously described [12]. Briefly, the number of podocyte foot processes per 10 µm glomerular basement membrane was determined in 2–5 places of each glomeruli, n = 15 glomeruli for all groups.

### Western Blotting

Protein was prepared from isolated glomeruli and a previously described mouse podocyte cell line [27]. Protein from a human melanoma cell line, A375 (ATCC, Manassas, VA), served as a positive control for MC1R expression. The protein samples were equally loaded (10 µg each) and western blotting was performed as previously described [12]. The following antibodies and dilutions were used for detection of specific proteins: anti-MC1R antibody 1∶500 (AMR-020, Alomone Labs, Ltd., Israel) and anti-actin goat polyclonal antibody 1∶1000 (sc-1615, Santa Cruz Biotechnology, Inc., Dallas, TX). An MC1R control peptide antigen (Alomone Labs, Ltd., Israel) was separately added at a 1∶1 weight ratio to the MC1R antibody solution before incubation to confirm antibody specificity.

### Statistical Analyses

All results are presented as mean ± SEM if not otherwise stated. Differences were determined using the Student’s *t*-test, or for morphological analyses one-way ANOVA. Differences in albuminuria between groups were determined with the exact Mann-Whitney non-parametric test, p<0.05 was considered statistically significant.

## Results

### MC1R Agonists Reduce Albuminuria in Experimental MN, but not in FSGS

#### Rats with PHN

**Figure 1:**
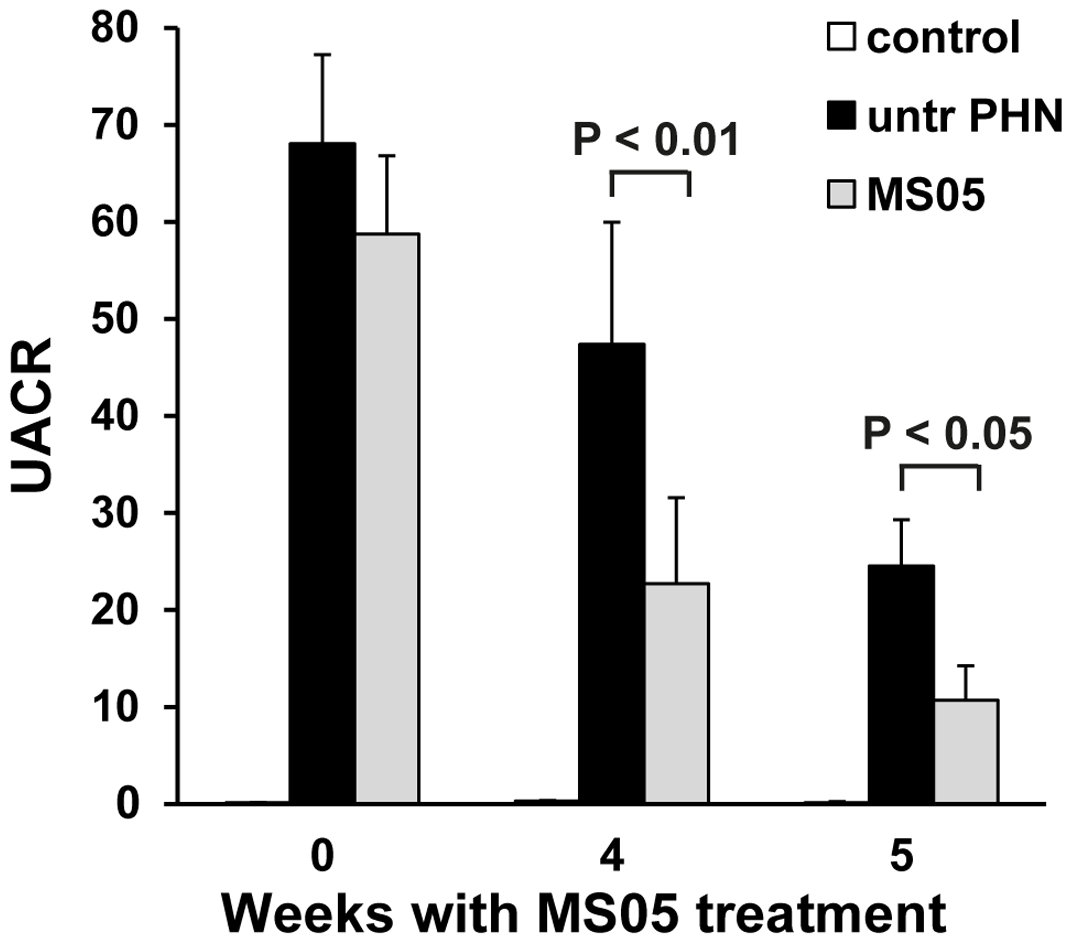
Albuminuria was reduced in MC1R agonist treated PHN rats. After four weeks of MS05 treatment (n = 17), the urinary albumin- to-creatinine ratio (UACR) was significantly reduced compared to untreated PHN (n = 14; p<0.01). One week after treatment withdrawal (week 5), albuminuria was further reduced in MS05-treated (n = 17) compared to untreated PHN rats (n = 13; p<0.05). Results are presented as geometrical mean ± SEM.

As can be seen in Figure 1, four weeks of treatment with the MC1R agonist MS05, gradually decreased the urinary albumin-to-creatinine ratio (UACR) from 58.7±8.1 to 22.7±8.8 (n = 17), compared to untreated PHN: from 68.0±9.2 to 47.4±13 (n = 14, p<0.01). Control animals remained at low and stable levels throughout the study: with UACR values below 0.4 (n = 5). In addition, one week after treatment withdrawal, the effect was sustained in MS05-treated rats with a UACR of 10.7±3.5 (n = 17), compared to untreated PHN rats: 24.5±4.8 (n = 13, p<0.05), corresponding to a 56% reduction. Within the MS05 group this corresponds to a 72±4.7% decrease in the level of albuminuria when comparing end of treatment with the peak level of albuminuria at treatment start (n = 17, p<0.001).

The weight of the rats was followed throughout the study of experimental MN and there was no significant difference in the weight between the groups at any point of the experiment.

#### Mice with adriamycin-induced FSGS

**Figure 2:**
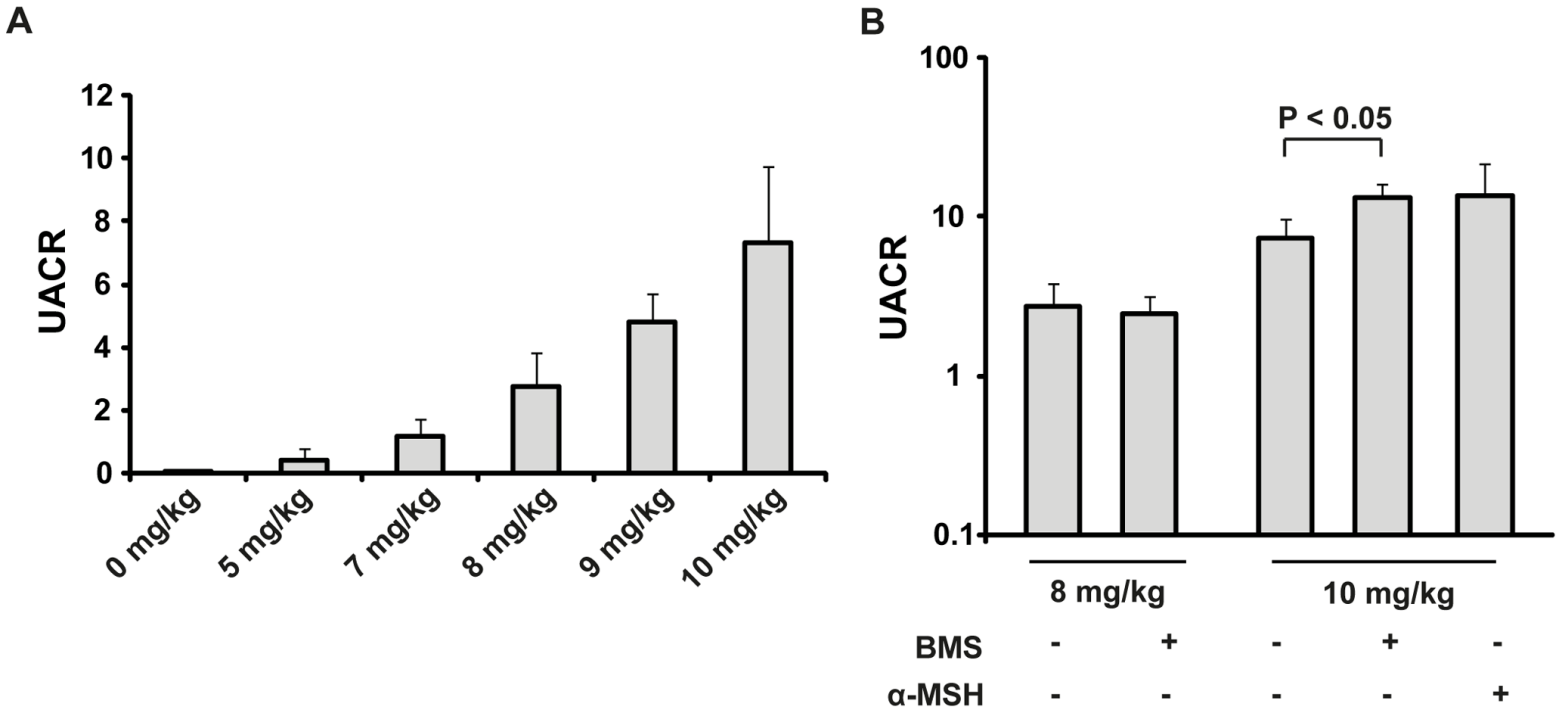
MC1R agonists did not reduce albuminuria in adriamycin-treated mice. (A) Five different doses of adriamycin, in the range of 5–10 mg/kg, were intravenously injected and the level of albuminuria was measured after 7 days. The urinary albumin-to-creatinine ratio (UACR) increased with increasing levels of adriamycin. (B) Albuminuria at day 7. Treatment with the MC1R agonist BMS-470539 did not reduce the level of albuminuria for the 8 mg/kg adriamycin dose, on the contrary it slightly increased albuminuria for the 10 mg/kg dose (n = 17–19, p<0.05). The unspecific melanocortin receptor agonist α-MSH did not have any significant effect (n = 9, n.s.) compared with untreated adriamycin mice. Results are presented as mean ± SEM.

Because of the narrow dose interval in adriamycin-induced nephropathy, a dose-response study was performed to ensure the optimal dose in this set-up. As can be seen in Figure 2A, the lowest dose of 5 mg/kg had UACR levels slightly increased compared to controls: 0.442±0.34 and 0.023±0.002, respectively. UACR was increased by the 7 mg/kg dose to 1.17±0.54, but with a large variation within the group and not high enough to ensure an established disease. Two of the higher doses were therefore picked for further treatment studies: 8 mg/kg, and 10 mg/kg.

To further explore the role of MC1R in nephrotic disease, the highly selective, synthetic, and stable agonist BMS-470539, and the unselective MCR agonist α-MSH were used for treating adriamycin-induced nephropathy (Figure 2B). For 8 mg/kg there was no difference in the level of albuminuria compared with the untreated mice. Thus, untreated mice subjected to adriamycin had a UACR of 2.75±1.1 (n = 19), compared to mice treated with the MC1R agonists BMS-470539; 2.46±0.73 (n = 19, n.s.). For the higher adriamycin dose, 10 mg/kg, that induced a more severe disease, BMS-470539 treatment resulted in a slightly higher UACR, 13.2±2.8 (n = 18) compared with untreated adriamycin mice, 7.34±2.4 (n = 17, p<0.05). Treatment with α-MSH did not significantly increase UACR, 13.6±7.9 (n = 9, n.s.). Controls remained at normal levels, i.e. UACR <0.02 (n = 10). At day 12 after adriamycin injection, the level of UACR had increased further, but with no differences between the groups: untreated adriamycin mice, 12.2±1.9 (n = 7); BMS-470539, 18.7±2.4 (n = 9, n.s.); α-MSH, 15.9±3.2 (n = 6, n.s.).

**Figure 3:**
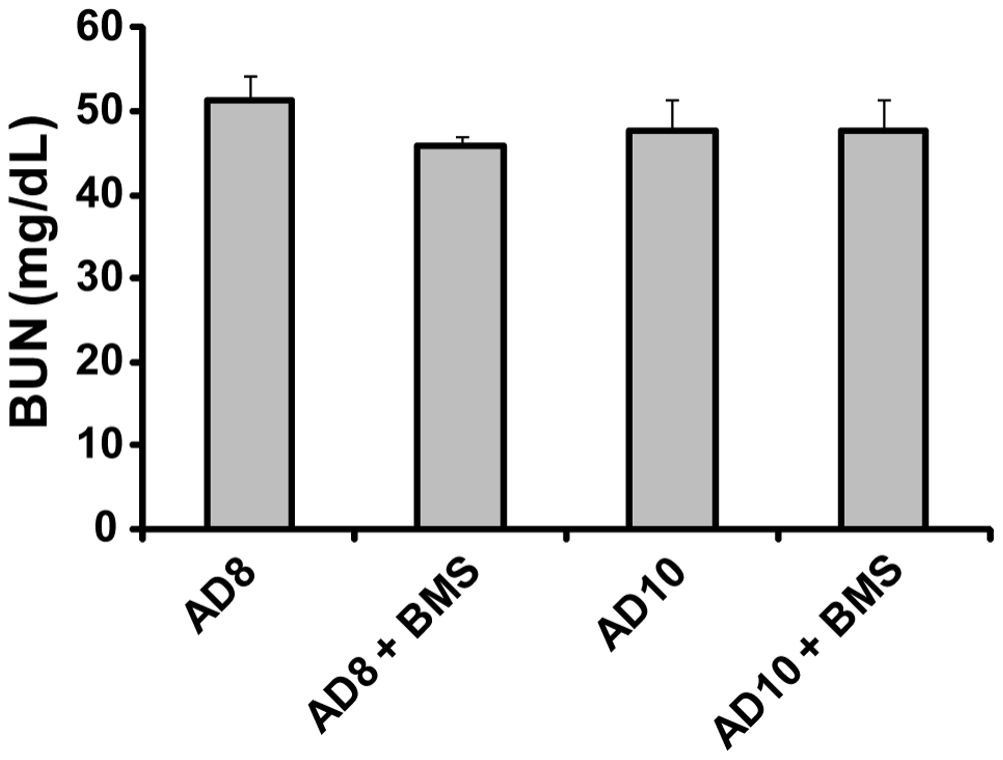
Blood urea nitrogen (BUN) does not differ in untreated adriamycin mice compared with MC1R agonist treated mice. BUN was measured in plasma samples taken from mice 8–10 days after injection of adriamycin. There was no difference between the groups (n = 9–10, n.s.). Results are presented as mean ± SEM.

Blood urea nitrogen (BUN) is commonly used to assess kidney function. As can be seen in Figure 3, there was no difference between mice with untreated adriamycin-induced nephropathy compared with those treated with BMS-470539 (n.s.).

Weight loss in adriamycin-treated mice was low due to daily intraperitoneal injections of a glucose-salt solution. At day 7, adriamycin-treated mice had lost approximately 5% of their initial body weight (p<0.05). There were no differences between the MC1R agonist treated groups and the vehicle-treated mice given adriamycin (n.s.).

### Podocyte Morphology is Improved by MC1R Agonists in MN but not in FSGS

**Figure 4:**
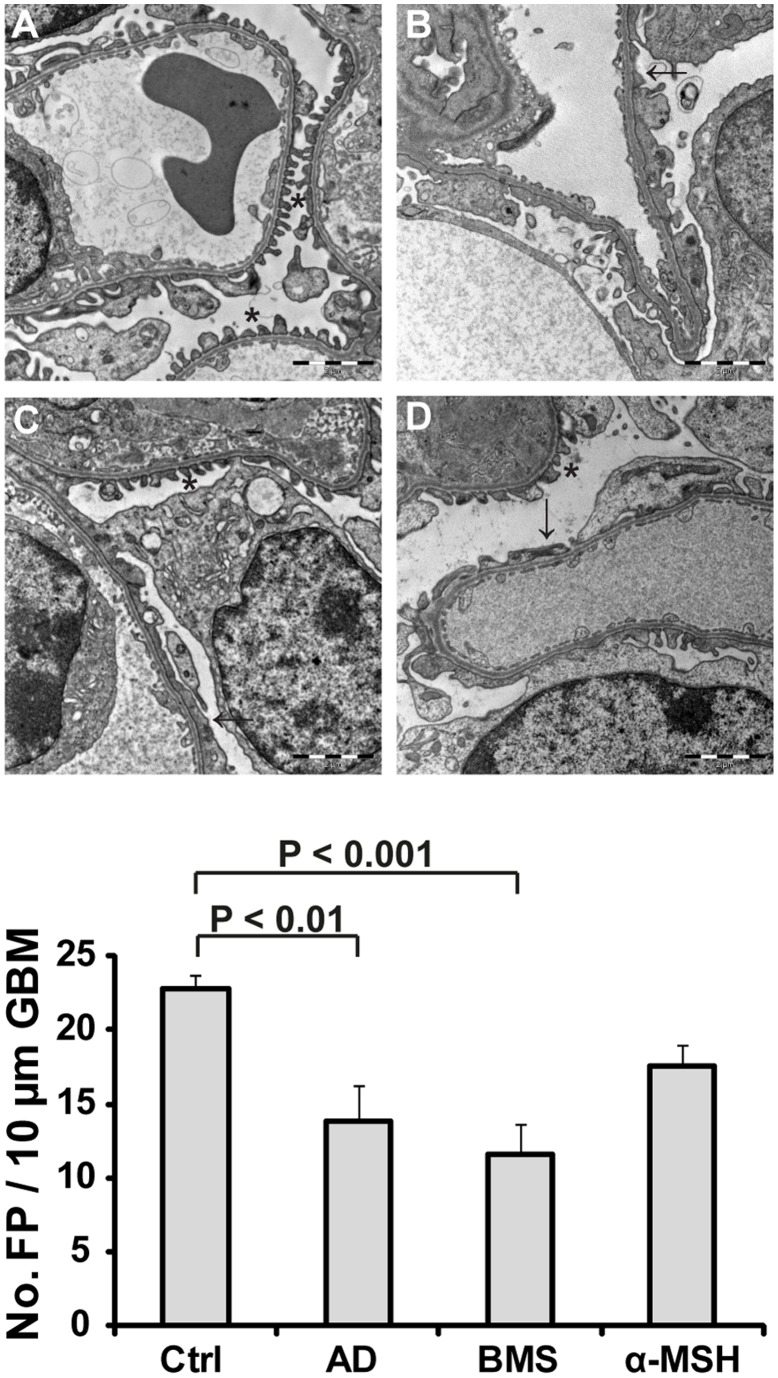
Glomerular morphology was disrupted in adriamycin treated mice. Kidneys were collected for morphological analysis at day 12 after adriamycin induced nephropathy. Slides were blindly examined by a pathologist (n = 15 for all groups). Shown are representative images of (A) controls, which present a normal glomerular structure and foot processes, (B) untreated adriamycin mice, displaying a high degree of foot process effacement, (C) treatment with BMS-470539 or (D) α-MSH, showing disrupted glomerular structures similar to untreated adriamycin mice. (E) The number of podocyte foot processes per 10 µm of glomerular basement membrane was quantified. Untreated adriamycin (AD) and mice treated with BMS-470539 (BMS) or α-MSH had a lower number of foot processes compared to controls (Ctrl). *Normal foot process, → disrupted glomerular barrier structure and loss of foot processes. Scale bar = 2 µm. FP = foot processes, GBM = glomerular basement membrane.

Treatment with the MC1R agonist MS05 improved morphology in the PHN rats [12]. Adriamycin treated mice displayed pathological changes with a focally disrupted glomerular barrier, including pronounced foot process effacement (Figure 4B). This effect was segmental and in line with the disease model, since FSGS nephrotic mice presented unaffected glomerular parts as well. Glomerular damage was also quantified by counting the number of foot processes along the glomerular basement membrane. Controls had 22.8±0.90 numbers of foot processes/10 µm glomerular basement membrane and displayed normal glomerular morphology (Figure 4A). Treatment with MC1R agonists did not improve, nor repair the disrupted glomerular structures (Figure 4C and 4D). Untreated adriamycin mice, BMS-470539 and α-MSH all had a lower number of foot processes compared to controls: 13.8±2.4 (p<0.01), 11.7±2.0 (p<0.001) and 17.6±1.4 (n.s.).

### Glomerular Expression of MC1R in Rat and Mouse

We have previously shown with real-time PCR that MC1R mRNA is expressed in human and rat kidney tissue with a C_T_ level of around 28 [12]. In this study, glomerular MC1R gene expression in PHN rats was confirmed but, as can be seen in Table 1, the C_T_ levels were higher (33) reflecting lower expression levels (Table 1). There was no change in expression between controls, MS05 treated and untreated PHN rats (n.s).

**Table 1:** Gene expression of MC1R in human, rat and mouse kidney tissue.

Species	Group	n	MC1R C_T_level	GAPDH C_T_level	2^∧^–(ΔΔC_T_)
Human*		1	28.9	21.1	
Rat†	Control	4	34.4±0.50	19.8±0.53	1
	Untreated PHN	11	33.3±0.37	20.5±0.20	0.94±0.26
	MS05	14	33.1±0.32	20.5±0.24	0.92±0.22
Mouse†	Control	10	33.5±0.37	16.6±0.08	1
	Adriamycin	7	33.2±0.28	16.9±0.21	0.86±0.19
	BMS-470539	8	33.3±0.27	16.7±0.18	1.0±0.35
	α-MSH	6	33.3±0.81	16.6±0.07	2.1±1.6

* Whole kidney tissue, previously published in Lindskog et al. [12].

† Glomeruli.

The mRNA expression levels in mice glomeruli were analyzed with real-time PCR (Table 1). The gene was expressed at low levels in mice with a mean C_T_ level of 33. There was no change in expression between the different groups (n.s).

**Figure 5:**
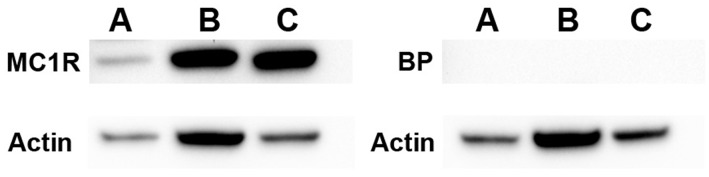
MC1R protein expression. Western blotting was performed in order to detect MC1R protein expression in mouse and human tissue. Left panel: incubation with a rabbit polyclonal MC1R antibody (Alomone Labs). Right panel: incubation with MC1R antibody and control blocking peptide antigen (BP) in order to show antibody specificity. A) mouse whole glomerular lysate, B) cultured wild type mouse podocytes and C) a human malignant melanoma cell line A375. MC1R protein expression was detected at the expected size of 37 kDa (left panel).

Protein expression of MC1R was also confirmed in mouse by western blotting, both in glomerular tissue as well as in a mouse podocyte cell line (Figure 5). A375, a human malignant melanoma cell line was used as positive control.

## Discussion

We have confirmed our previous findings that MC1R agonists can ameliorate passive Heymann nephritis mimicking human membranous nephropathy (MN). We also evaluated the role of MC1R in adriamycin-induced FSGS and found a different pattern: MC1R agonists did not reduce albuminuria in the FSGS-model, and did not improve podocyte morphology.

In our previous paper we showed that MC1R agonist treatment could ameliorate experimental membranous nephropathy in rats [12]. In the same study we demonstrated human MC1R mRNA expression in all glomerular cell types and MC1R protein expression specifically in the glomerular cell type called podocyte. Together, the human expression data and experimental data in rats propose a role for MC1R agonists in treating glomerular disease.

In this study, we have further evaluated the potential role of MC1R as a new treatment option.

In PHN rats treated with an MC1R agonist, the amelioration of albuminuria was sustained after treatment withdrawal, which is in line with clinical observations where albuminuria has been further reduced after removal of ACTH treatment [9], [10], [12]. These results suggest that the disease is reversible and that MC1R agonists affect the podocytes in a beneficial manner. Clinical data suggest that ACTH and melanocortin receptor agonists (MCRs) could ameliorate nephrotic disease [9]–[11]. Indeed, we have shown that MC1R co-localizes with synaptopodin, indicating specific expression in podocytes [12], which suggests a general role for the receptor in podocytopathic nephrotic diseases such as MN.

On the other hand, in experimental FSGS, specific MC1R agonists did not reduce albuminuria. There are possible explanations to this finding including different disease mechanisms between MN and FSGS, as well as species diversity in MC1R expression and function. The experimental models used mimic two different human glomerular diseases, with similar changes in podocyte appearance including loss of foot processes, but also with distinct morphological differences. In experimental FSGS, not only the podocytes, but also the glomerular endothelium, is dysfunctional [20] [28], [29]. Thus, the glomerular endothelial cell surface layer is markedly reduced in thickness after the administration of adriamycin [20]. Indeed, the endothelial changes precede those in the podocyte [29]. In FSGS, the complex signaling between podocytes and endothelial cells, required for the maintenance of an intact glomerular barrier, seems to be disturbed [28]. In contrast, the pathophysiological mechanisms behind MN involve immune-complexes, antibodies to megalin in Heymann nephritis and PLA2R in humans. These differences may explain why MC1R agonists are more or less effective. Another explanation for the discrepancy between the two disease models could be that the adriamycin-induced FSGS-like disease develops rapidly, while clinical FSGS has a slower time course. We showed that MC1R is present in not just human kidney tissue but also in rat and mouse. The MC1R agonists are highly effective for the human MC1R. However, it has been reported that mouse MC1R does not respond as well to stimulation with some of these agents as human MC1R [30], and the homology between human and murine MC1R is only 76% [31]. Thus, several aspects need to be considered when interpreting diverse effects of MC1R agonists on albuminuria in the different experimental disease models.

In summary, MC1R agonists reduce albuminuria and improve morphology in experimentally induced MN whereas they have no effect in experimental FSGS. The results illustrate the differences in these podocytopathies in terms of signaling mechanisms underlying proteinuria, and progression of disease. Further investigations of the mechanism behind MC1R signaling may reveal new information of how these diseases develop and could be treated.
